# Efficacy of Preoperative Administration of Paracetamol-Codeine on Pain following Impacted Mandibular Third Molar Surgery: A Randomized, Split-Mouth, Placebo-Controlled, Double-Blind Clinical Trial

**DOI:** 10.1155/2017/9246352

**Published:** 2017-02-23

**Authors:** Maria Paola Cristalli, Gerardo La Monaca, Chiara De Angelis, Nicola Pranno, Susanna Annibali

**Affiliations:** ^1^Department of Biotechnologies and Medical Surgical Sciences, Sapienza University of Rome, Rome, Italy; ^2^Department of Sense Organs, Sapienza University of Rome, Rome, Italy; ^3^Department of Oral and Maxillo-Facial Sciences, Sapienza University of Rome, Rome, Italy

## Abstract

*Objectives*. The aim of this study was to determine the effectiveness of preoperative administration of single-dose of paracetamol-codeine, in the relieving of acute postoperative pain after the surgical removal of an impacted mandibular third molar.* Materials and Methods*. The study cohort consisted of 32 Caucasian outpatients, giving a total of 64 bilateral symmetrical impacted mandibles. Patients were randomized in two experimental groups to receive a preoperative oral dose of paracetamol-codeine (analgesic group) or a placebo (placebo group) at the first and second surgeries. Study participants were asked to record pain intensity scores during the operation and the next 2 days, the time of the first request for rescue analgesic, and the total number of postoperative-supplement paracetamol-codeine tablets.* Results*. The pain intensity score on the first day was significantly lower in the analgesic group than in the placebo group (*p* < 0.001). The time to using rescue therapy was significantly longer in the analgesic group than in the placebo group (*p* = 0.004). The number of paracetamol-codeine tablets used postoperatively did not differ between the analgesic and placebo groups (*p* = 0.104).* Conclusions*. Preoperative paracetamol-codeine is effective in providing immediate postoperative pain control after third molar surgery and in delaying the initial onset of pain. This trial is registered with ClinicalTrials.gov Identifier (Registration Number): NCT03049878.

## 1. Introduction

The removal of impacted mandibular third molars is nowadays one of the most frequent oral surgical procedures. Nevertheless, postsurgical complications such as acute postoperative pain, swelling, bruising, and limited mouth opening occur frequently, and these have an immediate negative impact on the working and social lives of patients [1, 2]. The postoperative pain is undoubtedly the most unpleasant symptom for the patient and, despite receiving analgesic therapy, may persist even 1 week after the surgery, leading to increased morbidity and negatively influencing the well-being and quality of life [3–5].

Various analgesic combinations have been proposed for minimizing this complication. Combining different pharmacologic classes of analgesic drugs that have different mechanisms of action and side-effect profiles, resulting in additive or synergic effects, may improve the efficacy of pain therapy [6]. In addition, the use of the individual drugs at lower doses in such combinations is associated with a lower incidence of adverse effects [7, 8].

Furthermore, a single tablet containing two different analgesics at fixed doses seems to provide better relief of acute postoperative pain than the individual administration of the same drugs as separate tablets [9]. There are many reports in the literature of the effectiveness of combined paracetamol–codeine preparations for postoperative pain after the removal of wisdom teeth [8].

Paracetamol (also called acetaminophen) is a commonly prescribed analgesic used extensively in the management of acute pain, although its exact mechanism of action is not fully understood.

Paracetamol is absorbed well upon oral administration, reaching peak concentrations in 30–60 min, and its half-life is 2-3 hours. At a dosage of 4 g/day at 6-hour intervals, paracetamol is well tolerated in adults without compromising safety, although overdose has been linked to adverse events such as severe hepatotoxicity [7, 10].

Codeine (3-methylmorphine) is a mild opioid that is absorbed well upon oral administration with a weak analgesic effect [11]. The combination with paracetamol provides greater pain relief and is useful in controlling mild-to-moderate pain. The elimination half-life of codeine is 2.9 hours. Codeine at the adult normal dose (30–60 mg every 4 hours orally to a maximum of 240 mg daily) causes no euphoria or respiratory depression and is rarely addictive [7, 12].

The primary outcome of this randomized, split-mouth, placebo-controlled, double-blind clinical trial was the efficacy of preoperative administration of single-dose paracetamol-codeine in reducing pain intensity after the surgical removal of an impacted mandibular third molar under local anaesthesia. Additional analgesic efficacy outcomes included the number of patients using rescue therapy, time to the first use of rescue analgesia, total number of postoperative-supplement paracetamol-codeine tablets and the incidence of adverse effects occurring in each study group during the study period.

## 2. Materials and Methods

### 2.1. Study Design and Sample

This randomized, split-mouth, placebo-controlled, double-blind clinical trial was carried out at the Oral Surgery Unit, Policlinico Umberto I at “Sapienza” University of Rome, Italy, in accordance with standards of Good Clinical Practices for analgesic drugs and was approved by the local ethical committee (reference 2704/21.02.2013) in compliance with the 1964 Declaration of Helsinki on medical protocol and ethics and its later amendments or comparable ethical standards. Before giving their consents, all participants were informed of the possible risks associated with impacted mandibular third molar extraction and the aim of the experimental protocol.

Patient recruitment was conducted between April 2013 and September 2015 according to the CONSORT statement. The sample unit used in the present study was the surgical site. The study cohort consisted of Caucasian outpatients of both genders, aged between 20 and 29 years, selected to undergo elective surgical removal of bilateral symmetrical impacted mandibular third molars under local anaesthesia.

Subjects were enrolled using the following criteria: absence of systemic pathologies (ASA class I); nonsmoker; not pregnant or lactating; good oral hygiene; no drug consumption for 10 days before the operation; bilateral impacted mandibular third molars with comparable position, depth, and inclination; presence of the first and second molars; absence of painful symptoms and associated inflammatory or osteolytic pathologies in a radiographic examination; extraction difficulty index of >7 according to Pederson's scale [13] and a degree of IV (complex procedures) on a modified version of Parant's scale [14, 15].

Patients were randomized into two experimental groups: (1) preoperative oral dose of paracetamol-codeine (500-mg paracetamol + 30-mg codeine) (analgesic group) and (2) placebo (starch) (placebo group). One month after the first surgery, at complete wound healing, the analgesic and placebo groups underwent the second surgery for third molar extraction on the opposite side, but with the preoperative treatment inverted: those originally in the analgesic group received placebo and those originally in the placebo group received paracetamol-codeine. All of the operations were performed by the same experienced surgeon in order to minimize variations in the surgical technique.

### 2.2. Randomization

Presurgical analgesic treatment (paracetamol-codeine versus placebo) was assigned using a list of random numbers generated using CLINSTAT software (Martin Bland, York, UK) before the start of the study.

### 2.3. Blinding

The patients, surgeon, and statistician were all blinded to the type of presurgical treatment (double-blind procedure). The only nonblinded person was an external study collaborator involved in the verification of doses, who also had to record the code indicating to which group each patient had been assigned until the conclusion of the study.

### 2.4. Surgical Technique

Surgery was performed under local anaesthesia. The nerve block was achieved with 3% mepivacaine and the soft-tissue infiltration with 2% mepivacaine and 1 : 100,000 adrenaline (Carbocaine, AstraZeneca, Italy). A mucoperiosteal flap was raised by making an incision distal to the lower second molar along the length of the anterior border of the ascending ramus of the mandible. Ostectomy and tooth sectioning were then performed. After completely extracting the tooth, the socket was revised and the flap was sutured with interrupted synthetic nonabsorbable sutures (4/0 Ethilon™, Ethicon Ltd, UK). The surgery duration was measured from the start of the surgical procedure until completing the last suture. The sutures were removed 1 week later.

### 2.5. Study Medication

Professional oral hygiene was applied 7–10 days prior to surgery. One hour before the operation, all patients received antibiotic prophylaxis with 2-g amoxicillin + clavulanic acid (Augmentin, GlaxoSmithKline, Italy) to prevent postsurgical infection.

Fifteen minutes before surgery, one tablet containing either 500-mg paracetamol + 30-mg codeine (Coefferalgan, Farma 1000, Italy) or starch (placebo) was administered orally; the two types of tablets had an identical appearance. Immediately before applying local anaesthesia, all participants were instructed to execute three mouth rinses with an antiseptic at 0.20 mg/ml (0.2% chlorhexidine, Corsodyl, GlaxoSmithKline, Italy).

Postsurgical instructions were given to each patient concerning food consumption as well as hygiene at the surgical site (0.2% chlorexidine spray, Corsodyl, GlaxoSmithKline, Italy). During this period, both groups of patients (analgesic and placebo) were allowed to take the same paracetamol-codeine tablets given preoperatively at 6-hour intervals depending on their pain symptoms (up to a maximum of 4-g paracetamol and 240-mg codeine per 24 hours). Any adverse events (nausea, vomiting, headaches, or dizziness) occurring between the start of the trial and 1 week later at the time of suture removal were recorded.

### 2.6. Postoperative Pain and Rescue-Therapy Evaluation

The primary outcome of the study was the postoperative pain. Before surgery, patients were instructed to complete the Numerical Rating Scale-11 (NRS-11), which consisted of an interval scale ranging from 0 (“no pain”) to 10 (“maximum pain”) [16, 17]. All study participants were asked to record the pain intensity score at 1:00, 6:00, and 11:00 pm during the operative day and at 8:00 am, 1:00, 6:00, and 11:00 pm during the next two days.

Additional outcomes included the number of patients using rescue therapy, the time elapsed from the end of surgery until the first intake of analgesic medication, and the total number of paracetamol-codeine tablets with the same formulation taken by the patient in relation to the pain symptoms.

The effects on acute postoperative pain and rescue therapy were assessed only until the third day after surgery, since the onset of pain usually begins immediately after the local anaesthetic wears off, with a peak intensity after 6–8 hours and possibly persisting for a few days only [15, 18].

### 2.7. Statistical Analysis

The required sample size was calculated using statistics software (GPower 3.1.9.2, Heinrich-Heine-Universität, Düsseldorf, Germany) [19]. A power analysis using the Wilcoxon matched-pairs signed-rank test with an *α* level of 0.05 showed that 32 subjects would be adequate to obtain 80% power in detecting a statistical difference between 2 groups in scores on the NRS-11 for post-operative pain, assuming a loss to follow-up of 20%. The power calculation was based on the pain scores in a previous pilot study involving five patients (2.33 ± 2.00 [mean ± SD] for paracetamol-codeine and 2.92 ± 2.59 for placebo).

A database was created using Excel (Microsoft, Redmond, WA, USA), with appropriate checks used to identify errors. Descriptive statistics including mean and SD values were used. The Kolmogorov-Smirnov test was used to determine whether or not the data conformed to a normal distribution. The Wilcoxon signed-rank test was used to assess differences in pain intensity between the two groups. The paired* t*-test was used to compare between the two groups regarding the time of surgery, the time to the first use of rescue medication, and the amount of rescue analgesic taken. Fischer's exact test was used to assess the difference in the number of patients who required rescue therapy in the two groups. The Mann-Whitney test was used to evaluate the difference in the pain intensity between patients who took the analgesic drug in the first and second surgeries and between patients who took the placebo in the first and second surgeries. Data were evaluated using standard statistical analysis software (Statistical Package for the Social Sciences, version 20.0, IBM Corporation, Armonk, NY, USA). In each test the cut-off for statistical significance was *p* ≤ 0.05.

## 3. Results

In total, 32 patients were screened for study eligibility, comprising 17 females and 15 males with ages ranging from 20 to 29 years (22.65 ± 2.74 years), providing 64 surgical sites. There were no dropouts.

The duration of surgery did not differ between the analgesic (30.47 ± 12.03) and control (34.76 ± 12.21) groups (*p* = 0.202). No adverse events such as nausea, vomiting headaches, or dizziness were detected. The data are summarized in Table 1.

### 3.1. Pain Intensity Score

**Figure 1:**
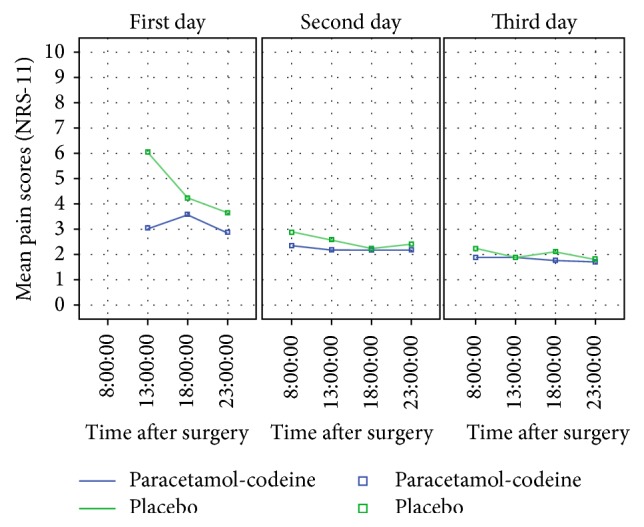
Pain intensity score (NRS-11) in the placebo and analgesic groups recorded at specific time intervals on the three days after surgery. Data are expressed as the mean and SD values. Significant difference in the NRS-11 score between the two groups was found only in the first day after surgery (*p* < 0.001).

The pain intensity score on the first day differed significantly between patients receiving the paracetamol-codeine combination (3.18 ± 2.18) and those receiving placebo (4.65 ± 2.73, *p* < 0.001). There was no difference on the second (*p* = 0.134) and third (*p* = 0.468) days after surgery. The data are illustrated in Figure 1.

The pain intensity score in the placebo group was significantly higher in the second surgery (5.48 ± 2.52) than in the first surgery (3.48 ± 2.62, *p* = 0.011). In contrast, the pain intensity score in the analgesic group did not differ significantly between the second (2.09 ± 2.02) and first (3.05 ± 1.75) surgeries (*p* = 0.419) (Table 2).

**Figure 2:**
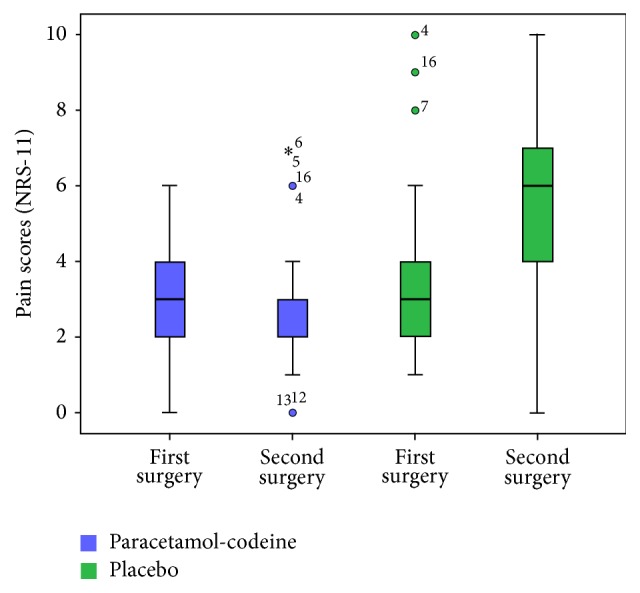
Box-and-whisker plots related to the pain scores (NRS-11) in patients which received the placebo or analgesic drug, respectively, in the first or second surgery. The dark line in the middle of the boxes is the median. The box represents the interquartile (IQ) range which contains the middle 50% of the records. The whiskers are lines that extend from the upper and lower edge of the box to the highest and lowest values which are no greater than 1.5 times the IQ range. The circles are outliers. These are defined as values that do not fall in the whiskers. The asterisks or stars are extreme outliers. These represent cases that have values more than three times the height of the boxes.

The pain intensity was much higher in patients who received the preoperative analgesic drug in the first surgery and the placebo in the second surgery than in patients who received placebo in the first surgery and the preventive analgesic drug in the second surgery (Figure 2).

### 3.2. Rescue Therapy

Six of the 32 patients did not request rescue therapy for either the first or second surgeries, and 2 required rescue therapy only after treatment with placebo. The difference between the two groups was not statistically significant (*p* = 0.364).

The mean time to first using rescue therapy was significantly longer in the analgesic group (414.33 ± 131.26 minutes) than in the placebo group (288.67 ± 116.63 minutes, *p* = 0.004). The number of postoperative-supplement paracetamol-codeine tablets used did not differ between the analgesic (1.24 ± 1.82) and control (1.47 ± 1.66) groups (*p* = 0.104).

## 4. Discussion

This study was designed to assess the effect of preoperative administration of single-dose paracetamol-codeine on postoperative pain after the surgical removal of an impacted mandibular third molar under local anaesthesia.

Third molar surgery is considered a validated and widely used pain model for the clinical evaluation of analgesic efficacy, because the removal of bone is associated with acute moderate-to-severe postoperative pain [17].

Furthermore, this procedure has proven selectivity, with the ability to distinguish active from inactive (placebo) medications, and it allows randomized clinical trials since it is often performed bilaterally [17].

The choice of applying tablets containing 500-mg paracetamol + 30-mg codeine (Coefferalgan, Farma 1000) was based on their previously reported effectiveness in relieving postoperative pain after impacted mandibular third molar surgery [8]. The preoperative administration of paracetamol-codeine so that its level peaks at the end of the action of the local anaesthesia seems to provide an optimal analgesic effect [20] and effectiveness in preventing the production of prostaglandin, whose concentration in the area of injury becomes significant about 1 hour after surgery [8, 18, 21]. In addition, a lower dose of codeine in combination with paracetamol produces an additive analgesic effect without increasing the incidence of side effects, with an excellent safety profile [22, 23].

To the best of the authors' knowledge, this is the first study to assess the effect on postoperative pain of a single dose of 500-mg paracetamol + 30-mg codeine administered preoperatively in the surgical removal of an impacted mandibular third molar under local anaesthesia. A few studies have evaluated the efficacy of 30-mg codeine in combination with paracetamol, but with doses varying from 300 to 650 mg and with most of them focusing on postoperative administration or as an intervention during general anaesthesia [8, 24].

Our data indicated that the preoperative administration of paracetamol-codeine was effective in immediate postoperative pain control, but not in the second and third day after surgery, and in lengthening the time before requiring postoperative analgesia. The not statistically significant difference between the less amount of codeine paracetamol tablets taken in the analgesic group compared to the placebo group may be due to the relatively small number of participants The efficacy of this analgesic premedication was also confirmed by the different trend in the pain intensity scores for the first and second extractions between patients pretreated with paracetamol-codeine as compared to placebo. The patients who received the preoperative analgesic drug in the first surgery experienced much stronger pain in the second surgery performed under placebo compared to the patients who received the placebo and analgesic drug in the opposite order. This finding could be due to the experience at the second extraction being influenced by the experience during the first. That is, patients who experience the preventive administration of paracetamol-codeine at the first surgery would expect a similar experience in the second, and so their perception of pain is amplified. In contrast, patients pretreated with placebo at the first surgery experience less pain than expected at the second surgery due to the preventive effect of the preoperative administration of paracetamol-codeine.

The lack of adverse events in the present study should be interpreted with caution due to the relatively small number of participants. However, adverse events are rare and typically occur when a higher dose of paracetamol has been taken for long periods of time or when the intake of codeine exceeds 90 mg daily [8].

Our results are consistent with the findings of similar studies comparing single doses of 30-mg codeine plus 300-mg paracetamol against placebo, in which this combination was demonstrated to be effective in immediate postoperative pain control and in reducing the time before requiring postoperative analgesia [20, 23, 25].

## 5. Conclusion

The preoperative administration of paracetamol-codeine significantly reduced the intensity of postoperative pain and increased the time to the first request of rescue therapy compared to placebo. These observations indicate that enhancing the control of postoperative pain requires the analgesic therapy to be applied perioperatively, starting before the surgery, extending into the early postsurgical period and continuing up to 12–24 hours postoperatively. Delaying analgesic administration until the patient reports mild-to-severe pain results in unnecessary discomfort and may reduce the efficacy of the treatment.

## Figures and Tables

**Table 1:** Comparison of objective measurement data between the placebo and analgesic groups.

	Analgesic group	Placebo group	*p*
Surgery duration (min)	30.47 ± 12.03	34.76 ± 12.21	0.202
Pain intensity scores on the first day	3.18 ± 2.18	4.65 ± 2.73	0.001^*∗*^
Pain intensity scores on the second day	2.22 ± 1.88	2.53 ± 2.12	0.134
Pain intensity scores on the third day	1.81 ± 1.77	2.01 ± 2.31	0.468
Time to first using analgesic intake (min)	414.33 ± 131.26	288.67 ± 116.63	0.004^*∗*^
Total rescue therapy intake (no. of tablets)	1.24 ± 1.82	1.47 ± 1.66	0.104
No. of patients who took no rescue analgesic	8	6	0.364

Data expressed as the mean ± standard deviation or number of patients.

Asterisks indicate significant differences.

**Table 2:** Comparison of pain scores (NRS-11) between patients who received the analgesic drug in the first surgery and the second surgery and between patients who received the placebo in the first surgery and the second surgery.

	First surgery	Second surgery	*p*
Pain intensity scores in the analgesic group	3.05 ± 1.75	2.09 ± 2.02	0.419
Pain intensity scores in the placebo group	3.48 ± 2.62	5.48 ± 2.52	0.011^*∗*^

Data expressed as the mean ± standard deviation.

Asterisk indicates a significant difference.
